# The IL-4/IL-13 signaling axis promotes prostatic fibrosis

**DOI:** 10.1371/journal.pone.0275064

**Published:** 2022-10-06

**Authors:** Quentin D’Arcy, Mehrnaz Gharaee-Kermani, Alisa Zhilin-Roth, Jill A. Macoska

**Affiliations:** 1 Center for Personalized Cancer Therapy, The University of Massachusetts, Boston, Massachusetts, United States of America; 2 Department of Biology, The University of Massachusetts, Boston, Massachusetts, United States of America; Medical Center - University of Freiburg, GERMANY

## Abstract

**Background:**

Lower urinary tract symptoms (LUTS) are a costly and pervasive medical problem for millions of aging men. Recent studies have showed that peri-urethral tissue fibrosis is an untreated pathobiology contributing to LUTS. Fibrosis results from excessive extracellular matrix deposition which increases transition zone and peri-urethral tissue stiffness and compromises prostatic urethral flexibility and compliance, producing urinary obstructive symptoms. Inflammatory cells, including neutrophils, macrophages, and T-lymphocytes, secrete a medley of pro-fibrotic proteins into the prostatic microenvironment, including IFNγ, TNFα, CXC-type chemokines, and interleukins, all of which have been implicated in inflammation-mediated fibrosis. Among these, IL-4 and IL-13 are of particular interest because they share a common signaling axis that, as shown here for the first time, promotes the expression and maintenance of IL-4, IL-13, their cognate receptors, and ECM components by prostate fibroblasts, even in the absence of immune cells. Based on studies presented here, we hypothesize that the IL-4/IL-13 axis promotes prostate fibroblast activation to ECM-secreting cells.

**Methods:**

N1 or SFT1 immortalized prostate stromal fibroblasts were cultured and treated, short- or long-term, with pro-fibrotic proteins including IL-4, IL-13, TGF-β, TNF-α, IFNγ, with or without prior pre-treatment with antagonists or inhibitors. Protein expression was assessed by immunohistochemistry, immunofluorescence, ELISA, immunoblot, or Sircoll assays. Transcript expression levels were determined by qRT-PCR. Intact cells were counted using WST assays.

**Results:**

IL-4Rα, IL-13Rα1, and collagen are concurrently up-regulated in human peri-urethral prostate tissues from men with LUTS. IL-4 and IL-13 induce their own expression as well as that of their cognate receptors, IL-4Rα and IL-13Rα1. Low concentrations of IL-4 or IL-13 act as cytokines to promote prostate fibroblast proliferation, but higher (>40ng/ml) concentrations repress cellular proliferation. Both IL-4 and IL-13 robustly and specifically promote collagen transcript and protein expression by prostate stromal fibroblasts in a JAK/STAT-dependent manner. Moreover, IL-4 and IL-13-mediated JAK/STAT signaling is coupled to activation of the IL-4Rα receptor.

**Conclusions:**

Taken together, these studies show that IL-4 and IL-13 signal through the IL-4Rα receptor to activate JAK/STAT signaling, thereby promoting their own expression, that of their cognate receptors, and collagens. These finding suggest that the IL-4/IL-13 signaling axis is a powerful, but therapeutically targetable, pro-fibrotic mechanism in the lower urinary tract.

## Introduction

Lower urinary tract symptoms (LUTS) are a costly and potentially critical medical problem for millions of aging men. This spectrum disorder encompasses symptoms such as weak stream, nocturia, incomplete emptying and intermittent urination, all of which are indicative of lower urinary tract dysfunction (LUTD). If left untreated or treated ineffectively, LUTD can progress to bladder dysfunction, which can lead to urinary retention, recurrent UTI, bladder calculi, and, eventually, renal impairment [1–4]. LUTD is often, although not always, concomitant with BPH—a proliferative but nonmalignant enlargement of the prostate. Surgical ablation of prostate tissue and medical approaches including 5α-reductase inhibitors, α-adrenergic receptor antagonists, and PDE5 inhibitors improve urinary flow, but such treatments are not effective for all men, can produce adverse effects that result in discontinuation of the therapeutic regimen, and do not abrogate the risk for disease progression [1, 5]. The latter point bears attention, as recent work evaluating Medical Therapy of Prostatic Symptoms (MTOPS) transition zone biopsies showed that high collagen levels consistent with fibrotic changes in tissue architecture were significantly higher in peri-urethral tissues from men whose LUTS progressed under 5α-reductase inhibitor and α-adrenergic receptor antagonist combination therapy.

Previous work by the Marberger group reported that inflammatory infiltrate in transurethral (TUR) prostate tissues from men demonstrating clinical progression of LUTS comprised ~70% CD4+ (helper) T cells and ~15% macrophages [6]. They also reported that the secretory pattern of these CD4+ T-helper cells was consistent with that of both T-helper Type 1 (Th1) cells, which secrete IFNγ and IL-2, and Type 2 (Th2) cells, which secrete IL-4 and IL-13 [6, 7]. All of these inflammatory mediators are pro-fibrotic proteins [6–8]. Torrko et al. reported that high levels of infiltrating CD4+ T-helper cells (p = .002) and CD68+ macrophages (p = .01) in transition zone prostate biopsies sampled as part of the MTOPS study were significantly associated with clinical BPH progression in that study [9]. Taken together, these studies show that Th2 cells and macrophages are highly prevalent in peri-urethral prostate tissues of men who demonstrate progressive LUTS.

Other studies have shown that peri-urethral tissue fibrosis is an untreated pathobiology contributing to LUTD [10–15]. Fibrosis results from excessive extracellular matrix (ECM) deposition which increases transition zone and peri-urethral tissue stiffness and compromises prostatic urethral flexibility and compliance, producing urinary obstructive symptoms and LUTD. Fibrosis is consequent to chronic or unresolved inflammation associated with aging, metabolic syndrome, or urinary tract infection. Inflammatory cells, including neutrophils, macrophages, and Th2 cells, secrete a medley of pro-fibrotic proteins into the prostatic microenvironment, including some of those described above: IFNγ, TNFα, CXC-type chemokines, and interleukins [6–8]. Together, these studies suggest that high levels of T-cell and macrophage inflammatory infiltrate are coincident with elevated prostate stromal collagen secretion in the periurethral region of the prostate in men suffering from progressive LUTS.

Among the inflammatory mediators secreted by Th2 cells, IL-4 and IL-13 are of particular interest because they share a common JAK/STAT6 signaling axis that promotes fibrillar collagen secretion and accumulation in multiple organ systems (reviewed in [16], most notably in the lung [17, 18], the kidney [19, 20], and the liver [17, 21]. Based on the studies described above, we hypothesized that IL-4 and IL-13 may contribute to peri-urethral prostate ECM accumulation and voiding dysfunction in the lower urinary tract. The studies reported here show that IL-4 and IL-13 promote their own expression, that of their cognate receptors, and ECM components, even in the absence of immune cells. These studies suggest that the IL-4/IL-13 signaling axis is a powerful, but therapeutically targetable, pro-fibrotic mechanism in the lower urinary tract.

## Results

### IL-4Rα and IL-13Rα1 are up-regulated in peri-urethral prostate with high collagen content

**Fig 1:**
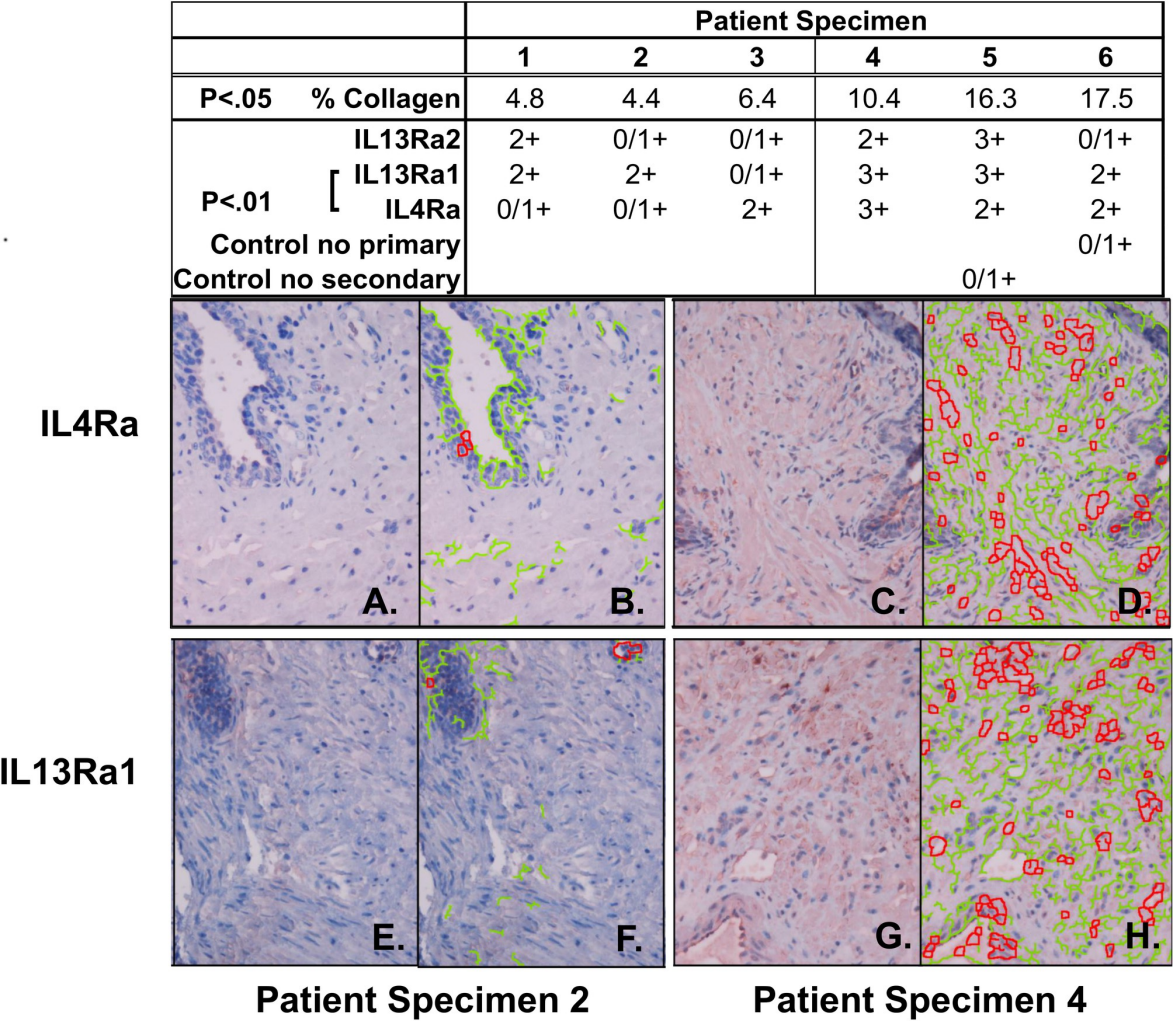
IL-4Ra and IL-13Ra1 are up-regulated in peri-urethral prostate with high collagen content. Tissue sections of peri-urethral prostate tissues from 6 patients were deparaffinized and assessed for collagen content using Masson’s Trichrome Stain [10] or for IL-4R, IL-13Ra1, and IL-13Ra2 protein expression by immunohistochemistry. Tissues from patients 4, 5 and 6 demonstrated significantly higher collagen contents than those from patients 1, 2 and 3 (p < .05) and significantly higher IL13Rα1 (p < .01) and IL4Rα (p < .01) protein expression levels. IL-4Rα staining is shown for patients 2 (**A, B**) and 4 (**C, D**), and IL-13Rα1 staining also for patients 2 (**E, F**) and 4 (**G, D**H. Panels **B, D, F** and **H** show the ImmunoRatio analysis of panels **A, C, E** and **G,** respectively, where green indicates incomplete/weak (0, 1+) and red indicates complete/strong (2+, 3+) staining (S1 Fig).

We have previously shown that high levels of collagen accumulation are associated with peri-urethral prostatic tissue fibrosis and lower urinary tracts symptoms (LUTS) [10, 22]. To determine whether collagen levels correlated with IL-4 and/or IL-13 receptor levels, peri-urethral tissue sections from 6 patients, 3 previously characterized with low 4.4–6.5% collagen content (patient specimens 1, 2, 3, Fig 1) and 3 with high 10.4–17.5% collagen content (patient specimens 4, 5, 6, Fig 1) [10] were subjected to immunohistochemistry to examine IL-4Rα, IL-13Rα1 and IL-13Rα2 expression levels (S1 Fig). IL-4Rα (p < .01) (Fig 1A–1D) and IL-13Rα1 (p < .01) (Fig 1E–1H) expression levels were significantly higher in tissues with high compared to low collagen content. Expression levels for the decoy receptor, IL-13Ra2, were heterogenous across the 6 patient sample set. These data show that IL-4Rα and IL-13Rα1 are significantly up-regulated concurrent with high collagen content in peri-urethral prostate tissues.

### Low doses of IL-4 and IL-13 enhance cellular proliferation

**Fig 2:**
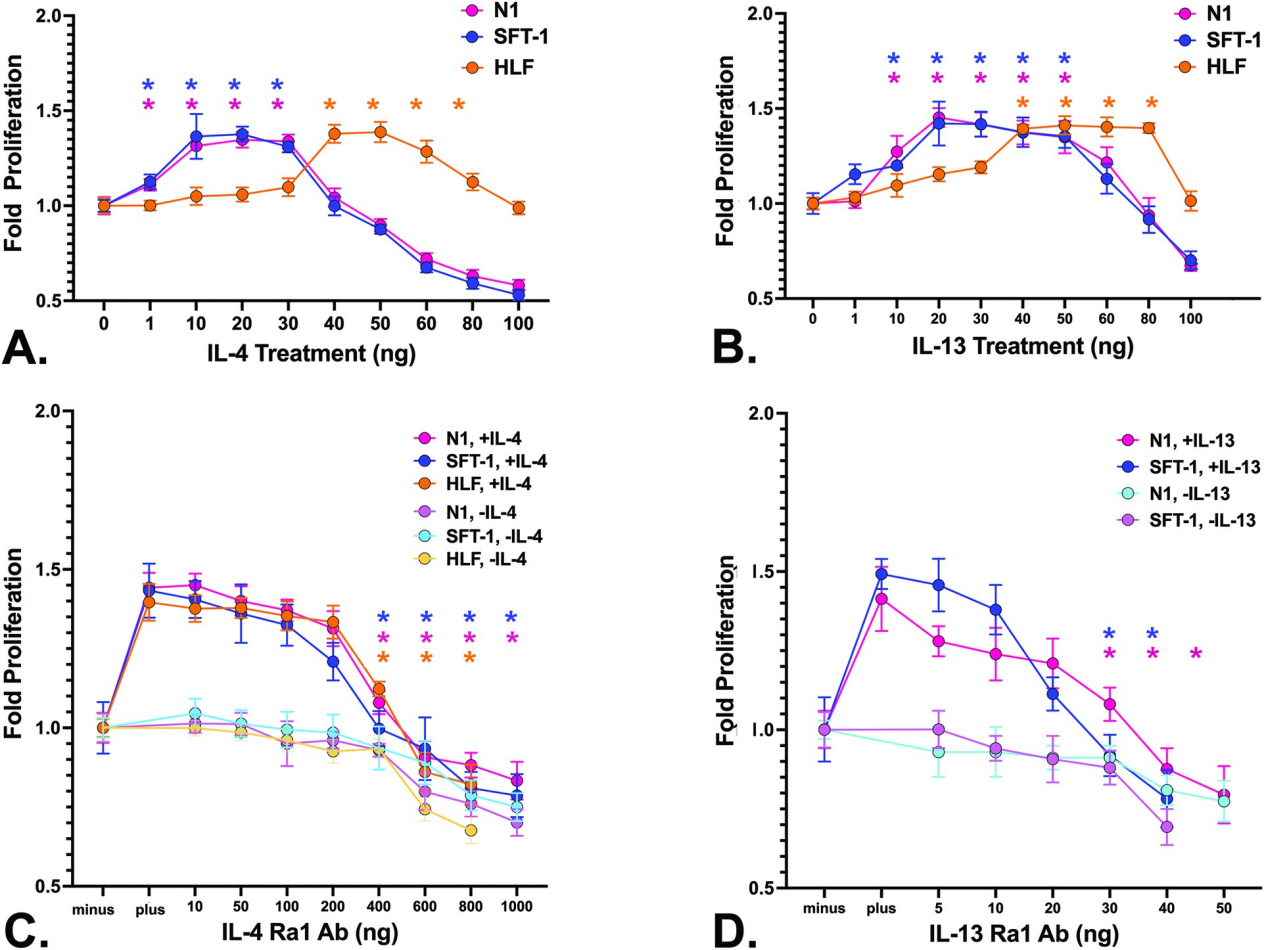
Low doses of IL-4 and IL-13 enhance cellular proliferation. **A, B.** N1 and SFT1 prostate fibroblasts and HLF primary lung fibroblasts were cultured in serum-free defined media supplemented with vehicle or increasing doses (1–100 ng) of human recombinant IL-4 (**A**) or IL-13 (**B**) for 24 hours. Proliferation was assessed by WST assay. Average cell numbers and standard deviations were calculated. Both IL-4 (**A**) and IL-13 (**B**) maximally stimulated the proliferation of N-1 and SFT-1 cells at a concentration of 20 ng/ml and of HLF cells at 40 ng/ml. These concentrations of IL-4 and IL-13, respectively, were therefore chosen for subsequent studies using N1, SFT-1, or HLF cells. Statistically (p < .05) increased proliferation levels compared to control are indicated as * in corresponding colors. **C, D.** N1 and SFT1 prostate fibroblasts and HLF primary lung fibroblasts were cultured in serum-free defined media, pre-treated for 2 hr with increasing doses of anti-IL-4Rα (10–1000 ng) (**C**) or IL-13Rα1 (5–50 ng) (**D**) antibodies, then cultured for 24 hr in the presence of 20 (N1, SFT-1 cells) or 40 (HLF cells) ng/ml IL-4 or IL-13, respectively. IL-4 failure to induce proliferation above basal levels (1-fold proliferation) was observed using 400 ng anti-IL-4Rαα (**C**), whereas IL-13 failure to induce proliferation above basal levels (1-fold proliferation) was observed using 40 ng anti-IL-13Rα1 (**D**). These concentrations of anti-IL-4Rα and anti-IL-13Rα1, respectively, were therefore chosen for subsequent studies using N1, SFT-1, or HLF cells (S2 Fig). Statistically (p < .05) decreased proliferation levels compared to control are indicated as * in corresponding colors.

IL-4 and IL-13 can act as cytokines to promote the proliferation of T-lymphocytes and multiple other cell types [23]. To determine whether these interleukins promoted fibroblast proliferation, N1 and SFT1 prostate fibroblasts and primary lung fibroblasts (HLF) (for comparison) were cultured in serum-free defined media supplemented with vehicle or increasing doses of human recombinant IL-4 and/or IL-13 (1–100 ng/ml) for 24 hours. These studies showed that both IL-4 (Fig 2A) and IL-13 (Fig 2B) significantly promoted prostate fibroblast proliferation at low concentrations, with maximal proliferation of 1.3–1.5X over untreated cells observed at 20 ng/ml (S2 Fig). Higher interleukin doses repressed proliferation. Interestingly, primary human lung fibroblasts achieved maximal proliferation of 2X at higher levels of IL-4 or IL-13 (40ng/ml, Fig 2A and 2B) (S2 Fig). Moreover, primary lung fibroblasts promoted maximal levels of cellular proliferation over a wide IL-13 concentration range spanning 40-80ng/ml (Fig 2B) (S2 Fig). These studies show that low, potentially physiological, levels of IL-4 or IL-13 promoted low-level proliferation of prostate fibroblasts, and that prostate fibroblasts were less responsive to the stimulatory effect of IL-4 and IL-13 compared to primary human lung fibroblasts.

To test the specificity of the IL-4/IL-13 induced proliferative response, cells were pre-treated with increasing doses of antibody against the IL-4Rα or IL-13Rα1 receptor, then stimulated with the concentration of IL-4 or IL-13 that produced the highest proliferative response. IL-4 failure to induce proliferation above basal levels (1-fold proliferation) was observed using 400 ng anti-IL-4Rα (Fig 2C) (S2 Fig), whereas IL-13 failure to induce proliferation above basal levels (1-fold proliferation) was observed using 40 ng anti-IL-13Rα1 (Fig 2D) (S2 Fig). These data suggest that IL-4 solicits a stronger, or perhaps more sustained, proliferative response than IL-13, as 10X higher IL-4Rα than IL-13Rα1 antibody levels were required to repress this response. Alternatively, this result may be due to differences in ligand/receptor affinities.

### IL-4 and IL-13 induce self- expression in prostate fibroblasts

**Fig 3:**
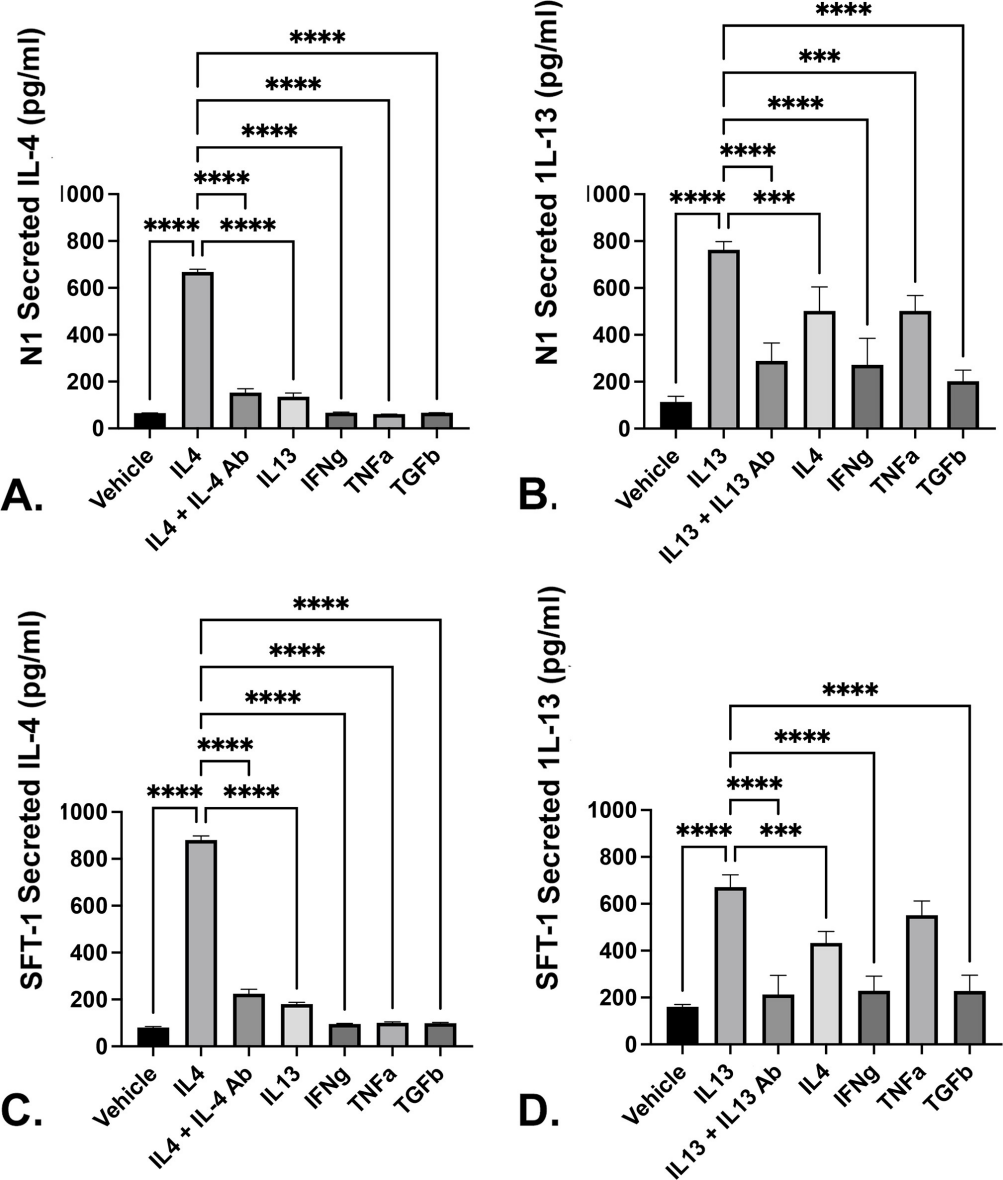
IL-4 and IL-13 induce self expression in prostate fibroblasts. N1 or SFT1 prostate fibroblasts were cultured in serum-free media supplemented with vehicle or 20ng/ml IL-4, IL-13, IFN-γ, TNFα, or 4ng/ml TGF-β, for 24 hours with or without 2 hr pre-treatment with 400 ng/ml IL-4Rα or 40 ng/ml IL-13Rα1 antibody. The cells were then washed, switched to fresh serum-free media, and incubated for another 24 hrs. Conditioned media was collected and interrogated by ELISA for IL-4 or IL-13 protein secretion (N1 cells, **A** and **B**; SFT1 cells, **C** and **D**) (S3 Fig), respectively. Statistically significant differences between IL-4 or IL-13 secretion by vehicle-treated compared to other treatments is indicated as * p < .05; ** p < .01; *** p < .001; **** p < .0001.

The IL-4/IL-13 signaling axis up-regulates and activates the STAT6 and GATA-3 transcription factors, which then promote their own expression as well as that of the IL-4 and IL-13 interleukins and their cognate receptors, IL-4Rα and IL-13Rα1 in immune cells (reviewed in [24]). Therefore, we investigated whether IL-4 and/or IL-13 promoted their own secretion by prostate fibroblasts. For these experiments, N1 or SFT1 prostate fibroblasts were cultured in serum-free media supplemented with vehicle or 20ng/ml IL-4, IL-13, IFN-γ, TNFα, or 4ng/ml TGF-β, for 24 hours with or without 2 hr pre-treatment with 400 ng/ml IL-4Rα or 40 ng/ml IL-13Rα1 antibodies, then switched to fresh serum-free media and incubated for another 24 hrs. Conditioned media was collected and interrogated by ELISA for IL-4 or IL-13 protein secretion. These studies showed that IL-4 induced its own secretion in both N1 and SFT 1 cells, and that this effect was ablated upon pretreatment with IL-4Rα antibody (Fig 3A and 3C). Parallel studies showed that IL-13 also induced its own secretion in N1 and SFT1 cells, which was ablated upon pre-treatment with IL-13Rα1 antibody (Fig 3B and 3D). IL-4 and IL-13 induced their own expression at levels significantly higher than that achieved by IFN-γ, TNFα, or TGFβ (Fig 3) (S3 Fig). Taken together, these data show that IL-4 and IL-13 can induce their own production by prostate fibroblasts. Moreover, whereas IL-4 induction was fairly specific, IL-13 induction was promoted by itself as well as other pro-fibrotics.

### IL-4 and IL-13 up-regulate collagen expression

**Fig 4:**
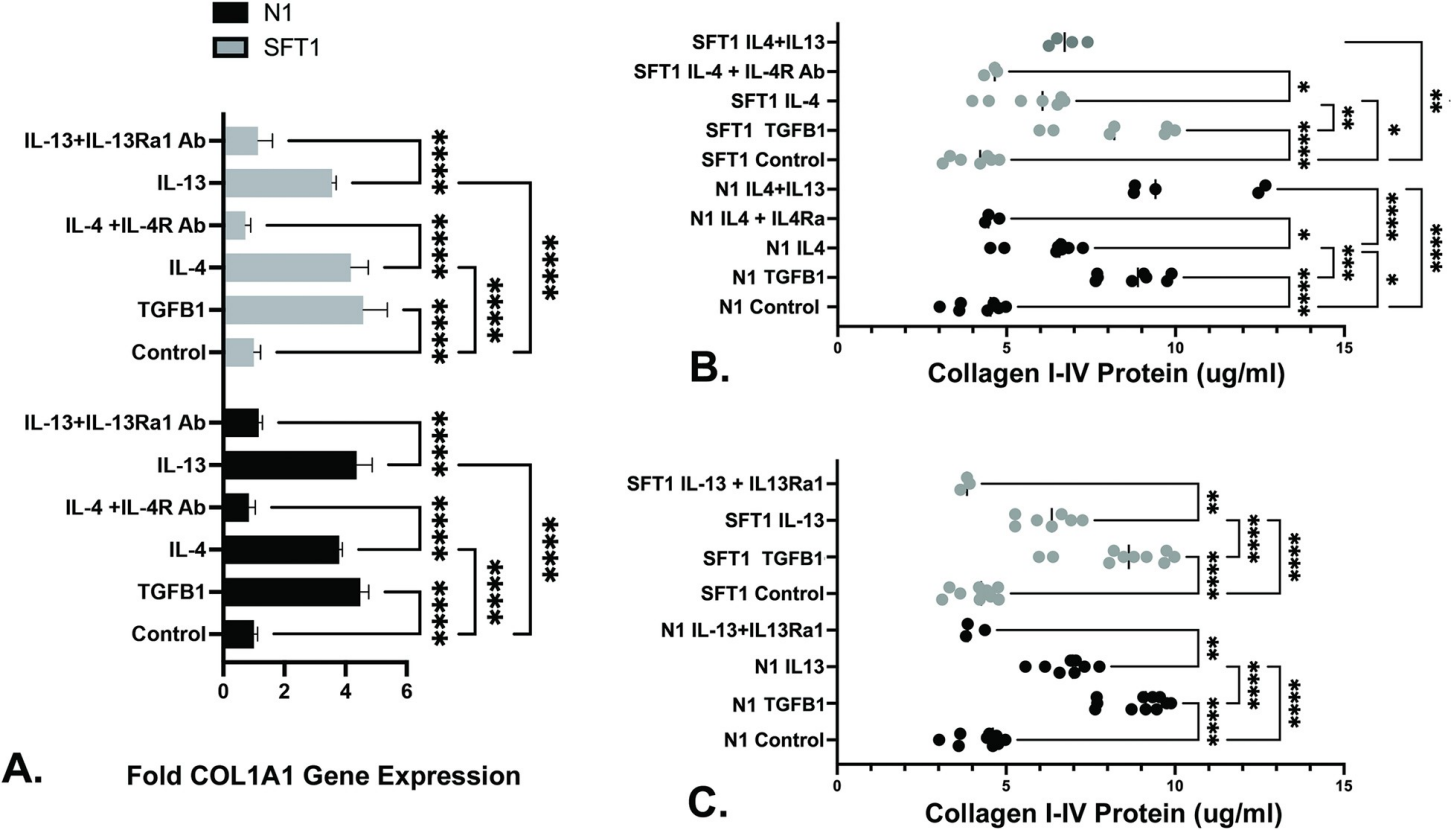
IL-4 and IL-13 increased collagen 1 gene transcription and collagen types I-V protein expression. **A.** Total RNA purified from N1 or SFT1 prostate fibroblasts cultured in serum-free media supplemented with vehicle or 20ng/ml IL-4, IL-13, or 4ng/ml TGF-β, for 24 hours with or without 2 hr pre-treatment with 400 ng/ml IL-4Rα or 40 ng/ml IL-13Rα1 antibody was subjected to qRT-PCR and assessed for transcription of the RPLPO (housekeeping) and COL1A1 genes. Cycle numbers to threshold were calculated by subtracting averaged untreated from averaged treated values and normalized to those of RPLPO. Transcript levels are expressed as fold changes over control. IL-4 and IL-13 induced COL1A1gene expression to levels 3-4x higher, and TGF-**β** to level 4-5x higher, than vehicle-treated (control) cells. Pre-treatment with antibody against IL-4Rα or IL-13Rα1 repressed COL1A1gene expression to near basal levels. Results shown represent the means of 3 experiments. Significant differences between paired comparisons are indicated as **** p < .0001. Error bars represent SD. Data used to generate graph provided in S4 Fig. **B, C.** N1 or SFT1 prostate fibroblasts were cultured in serum-free media supplemented with vehicle or 20ng/ml IL-4, IL-13, or 4ng/ml TGF-β, for 48 hours with or without 2 hr pre-treatment with 400 ng/ml IL-4Rα or 40 ng/ml IL-13Rα1 antibody. The cells were washed, lysed, and assessed for secreted soluble collagen types I-V using Sircol assay reagents. Both IL-4 (**B**) and IL-13 (**C**) significantly promoted collagen types I-V protein expression, which was ablated upon pre-treatment with IL-4Rα or IL-13Rα1 antibody, respectively (S4 Fig). Significant differences between paired comparisons are indicated as * p < .05; ** p < .01; *** p < .001; **** p < .0001.

IL-4 and IL-13 are pro-fibrotic proteins that can promote the secretion of ECM proteins [25]. We therefore investigated whether IL-4 and/or IL-13 could induce the expression of the COL1A1 gene, which encodes 2 of the 3 strands of the collage 1 protein trimer, in prostate fibroblasts. As seen in Fig 4A, IL-4 and IL-13 significantly increased COL1A1 gene expression to levels 3-4x higher than vehicle-treated N1 and SFT1 cells (p < .0001). TGF-**β** induced higher COL1A1 expression than IL-4 or IL-13, to levels 4-5x higher than vehicle-treated cells (p < .0001). Pre-treatment with antibodies against IL-4Rα or IL-13Rα1 ablated the observed IL-4 or IL-13-mediated COL1A1 transcriptional response, respectively, demonstrating the specificity of these responses (S4 Fig). Subsequent studies examined whether increases in COL1A1 transcript expression were mirrored by collagen protein expression. Sircol assays, which detect total soluble collagens I-V, demonstrated significantly higher levels of soluble collagens I-V produced by N1 and SFT-1 cells treated with IL-4 (p < .001) (**B**) or IL-13 (p < .0001) (**C**) compared to vehicle-treated cells. Pre-treatment with antibodies against IL-4Rα or IL-13Rα1 ablated the observed IL-4 or IL-13-mediated collagen protein expression to levels similar to those of vehicle-treated cells (p < .05). As expected, TGF-**β** induced higher levels of soluble collagens I-V than either IL-4 or IL-13. Co-treatment with IL-4 + IL-13 significantly increased production of soluble collagens I-V above those observed for IL-14 alone by N1 (p < .0001) and SFT-1 (p < .01) cells (Fig 4B). These data show that both IL-4 and IL-13 robustly and specifically promote collagen transcript and protein expression by prostate stromal fibroblasts to levels almost equivalent to those induced by the strong pro-fibrotic protein, TGF-**β** (S4 Fig).

### IL-4 and IL-3 do not promote αSMA expression

**Fig 5:**
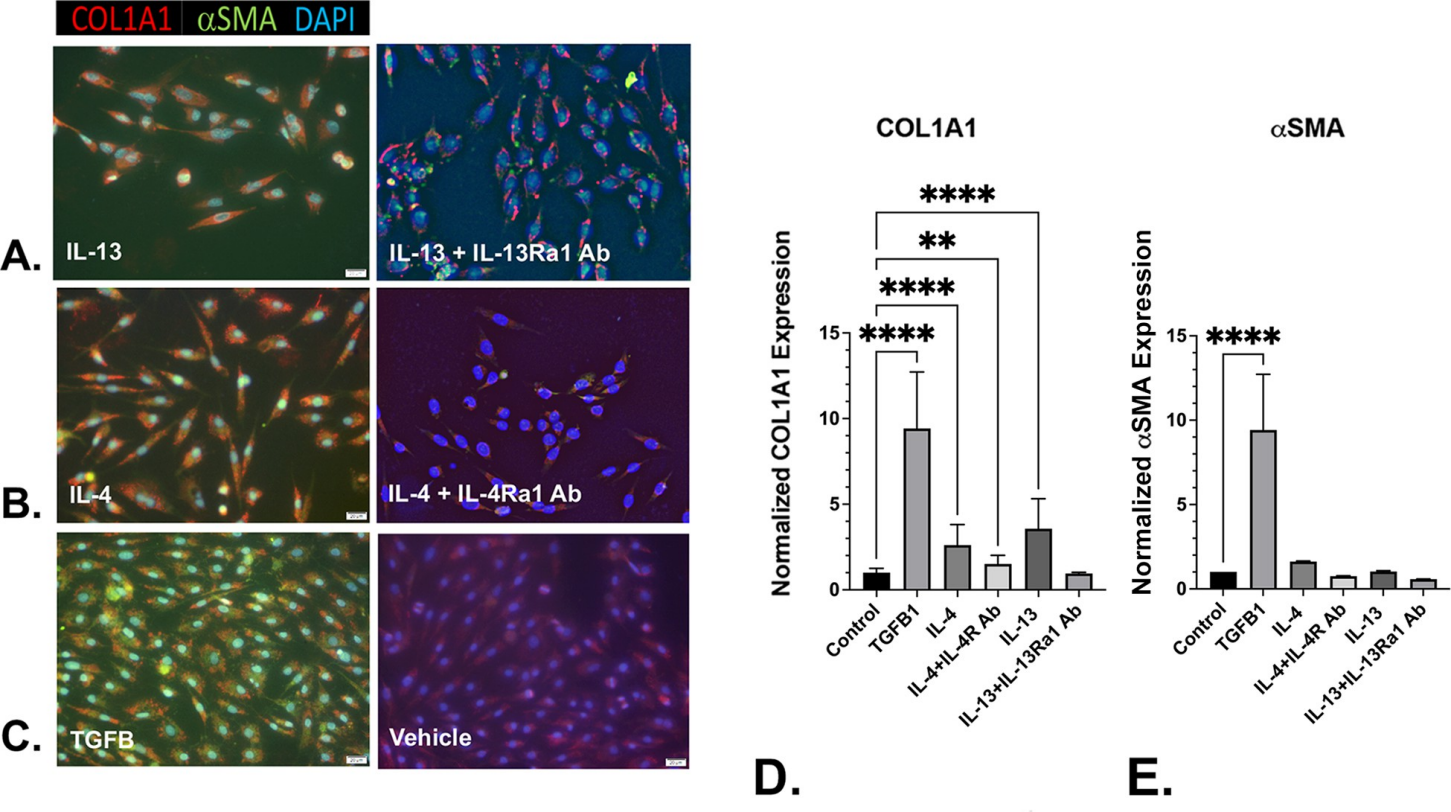
IL-4 and IL-13 promote COL1A1 and αSMA co-expression and upregulate receptors inhibitors repress IL-4 and IL-13-mediated prostate myofibroblast phenoconversion. N1 prostate fibroblasts were treated with 20 ng/ml of IL-13 or IL- 4, or 4 ng/ml TGFβ1 (positive control) for 48 hr, then co-immunostained for COL1A1 (PE-cy5-conjugated Ab, red), αSMA (fluorescein-conjugated Ab, green), or counterstained with nuclear-specific DAPI (blue), and the images merged. N1 cells treated with IL-13 (**A**), IL- 4 (**B**), or TGFβ1 (**C**), demonstrated significantly (**D**) increased expression COL1A1 *in vitro* compared to vehicle-treated cells, but only cells treated with TGFβ1 demonstrated significantly increased expression of αSMA. Pre-treatment with antibodies against the cognate receptors of IL-3 and IL-14, respectively, repressed the induction of COL1A1 protein expression (**A, B, D**), demonstrating the specificity of the cellular response to interleukin treatment. N1 cells treated with TGF-β1 exhibited a morphological change from a spindle-like shape to a more stellate shape consistent with the myofibroblast phenotype (**C**), whereas cells with IL-13 (**A**) or IL-4 (**B**) retained a spindle-like morphology suggesting a lack of myofibroblast differentiation. Images were captured and photographed using fluorescence microscopy on an EVOS® FL Auto system at 20X magnification. Fluorescence quantitation was assessed using Image-Pro Plus 7 imaging software. The number of cells evaluated for COL1A1 expression were 495 (control), 578 (TGFβ), 889 (IL-4), 224 (IL4+IL4R Ab), 761 (IL-13), and 96 (IL-13+IL-13Rα1 Ab). The number of cells evaluated for αSMA expression were 495 (control), 578 (TGFB), 56 (IL-4), 142 (IL4+IL4R Ab), 172 (IL-13), and 98 (IL-13+IL-13Rα1 Ab) (S5 Fig). Significant differences between paired comparisons are indicated as * p < .05; ** p < .01; *** p < .001; **** p < .0001.

N1 cells treated with TGF-β1, IL-4, or IL-13 demonstrated significantly increased expression of COL1A1 protein *in vitro* compared to vehicle-treated cells. (Fig 5A–5D). Pre-treatment with antibodies against the cognate receptors of IL-4 and IL-13, respectively, repressed their induction of COL1A1 protein expression (Fig 5A–5D), demonstrating the specificity of the cellular response to interleukin treatment. αSMA expression, however, was significantly upregulated by treatment with TGF-β1, but not with IL-4 or IL-13 (Fig 5A–5C and 5E) (S5 Fig). Moreover, N1 cells treated with TGF-β1 exhibited a morphological change from a spindle-like shape to a more stellate shape consistent with the myofibroblast phenotype (Fig 5C) [11], whereas cells treated with IL-4 or IL-13 retained a spindle-like morphology (Fig 5A–5C), suggesting that IL-4 and IL-13 induced fibroblast activation, but not complete differentiation to a myofibroblast phenotype.

### IL-4 and IL-13 induce expression of their cognate receptors *in vitro*

**Fig 6:**
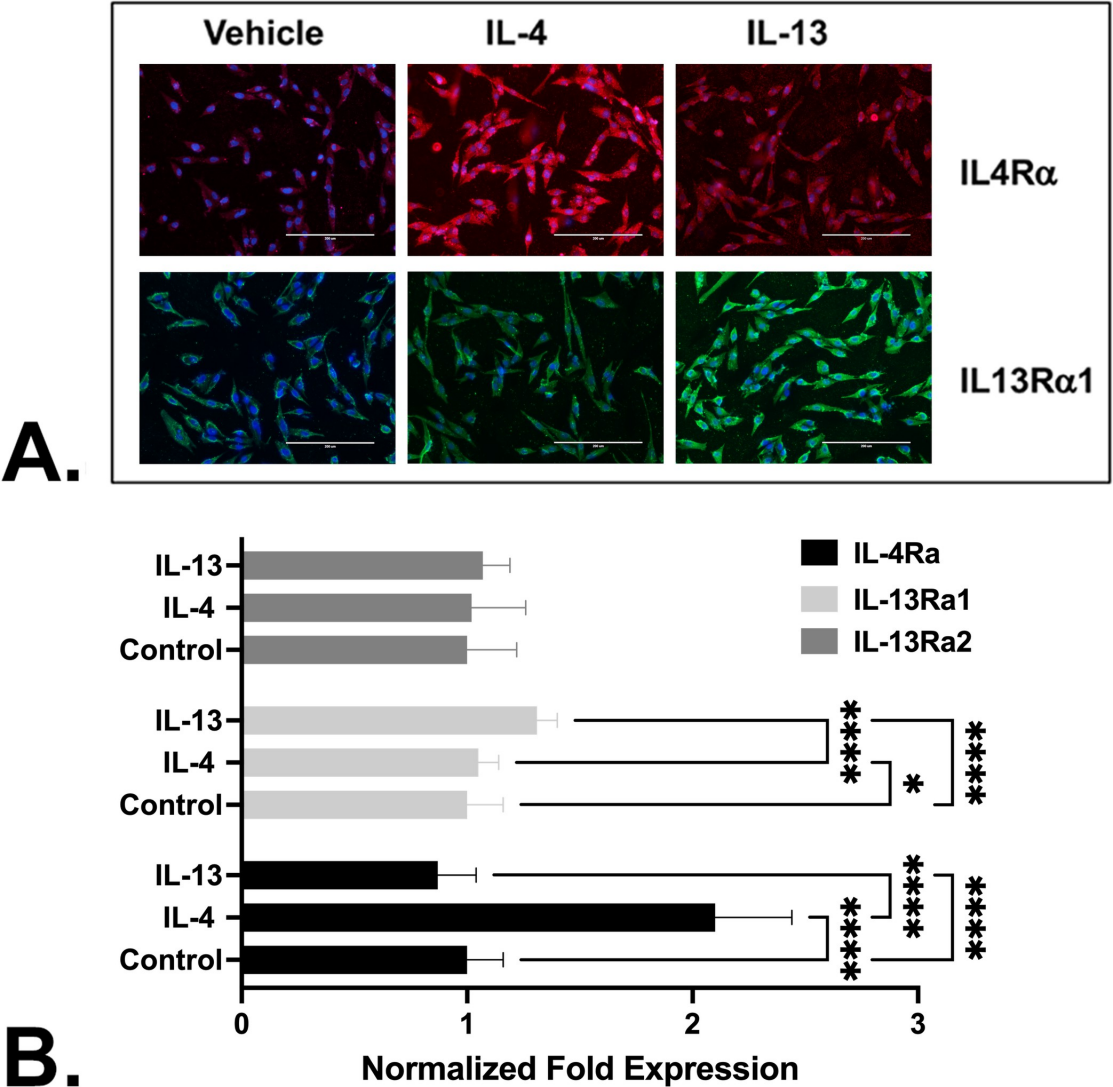
IL-4 and IL-13 induce expression of their cognate receptors *in vitro*. **A.** N1 prostate fibroblast cells were treated with vehicle, 20 ng/ml IL-13, or 20ng/ml IL- 4, for 48 hr, fixed, permeabilized, and immunostained using mouse monoclonal IL-4Rα primary antibody detected with goat anti-mouse Alexa Fluor 594 secondary antibody (red), or goat polyclonal IL-13Rα1 primary antibody detected with donkey anti-goat Alexa Fluor 488 secondary antibody (green). **B**. Quantitation of IL-4Rα or IL-13Rα1 in vehicle, IL-4, or IL-13 treated cells. Treatment with IL-4 or IL-13 significantly (p < .0001) up-regulated expression of their cognate receptors but not that of the decoy receptor, IL-13Rα2. The number of cells evaluated for IL4Rα expression were 101(control), 57 (IL-4), and 475 (IL-13). The number of cells evaluated for IL13Rα1 expression were 178 (control), 140 (IL-4), and 109 (IL-13). The number of cells evaluated for IL13Rα2 expression were 165 (control), 204 (IL-4), and 138 (IL-13) (S6 Fig). Significant differences between paired comparisons are indicated as * p < .05; ** p < .01; *** p < .001; **** p < .0001.

As noted above, The IL-4/IL-13 signaling axis up-regulates and activates the STAT6 and GATA-3 transcription factors, which then promote their own expression as well as that of the IL-4 and IL-13 interleukins and their cognate receptors, IL-4Rα and IL-13Rα1 in immune cells (reviewed in [24]). To begin to examine this in prostate fibroblasts, we first determined whether IL-4 and/or IL-13 induced the expression of their cognate receptors, IL-4Rα and IL-13Rα1. As shown in Fig 6A and quantitated in Fig 6B, N1 cells endogenously expressed both IL-4Rα and IL-13Rα1 under basal conditions. IL-4 significantly (p<0001) induced expression of IL-4Rα to levels ~3X higher than basal levels or those observed after IL-13 treatment. Conversely, treatment with IL-13 did not induce or repress IL-4Rα expression levels compared to basal expression (Fig 6B). Treatment with IL-13 was associated with a significant (p < .0001) increase in IL-13Rα1 expression levels above basal level, whereas IL-4 treatment had no effect on in IL-13Rα1 expression levels (Fig 6B). Neither IL-4 nor IL-13 induced or repressed expression of the IL-13Rα2 decoy receptor, which was unchanged from basal levels (Fig 6B) (S6 Fig).

### IL-4Ra drives IL-4- and IL-13-mediated signal transduction

**Fig 7:**
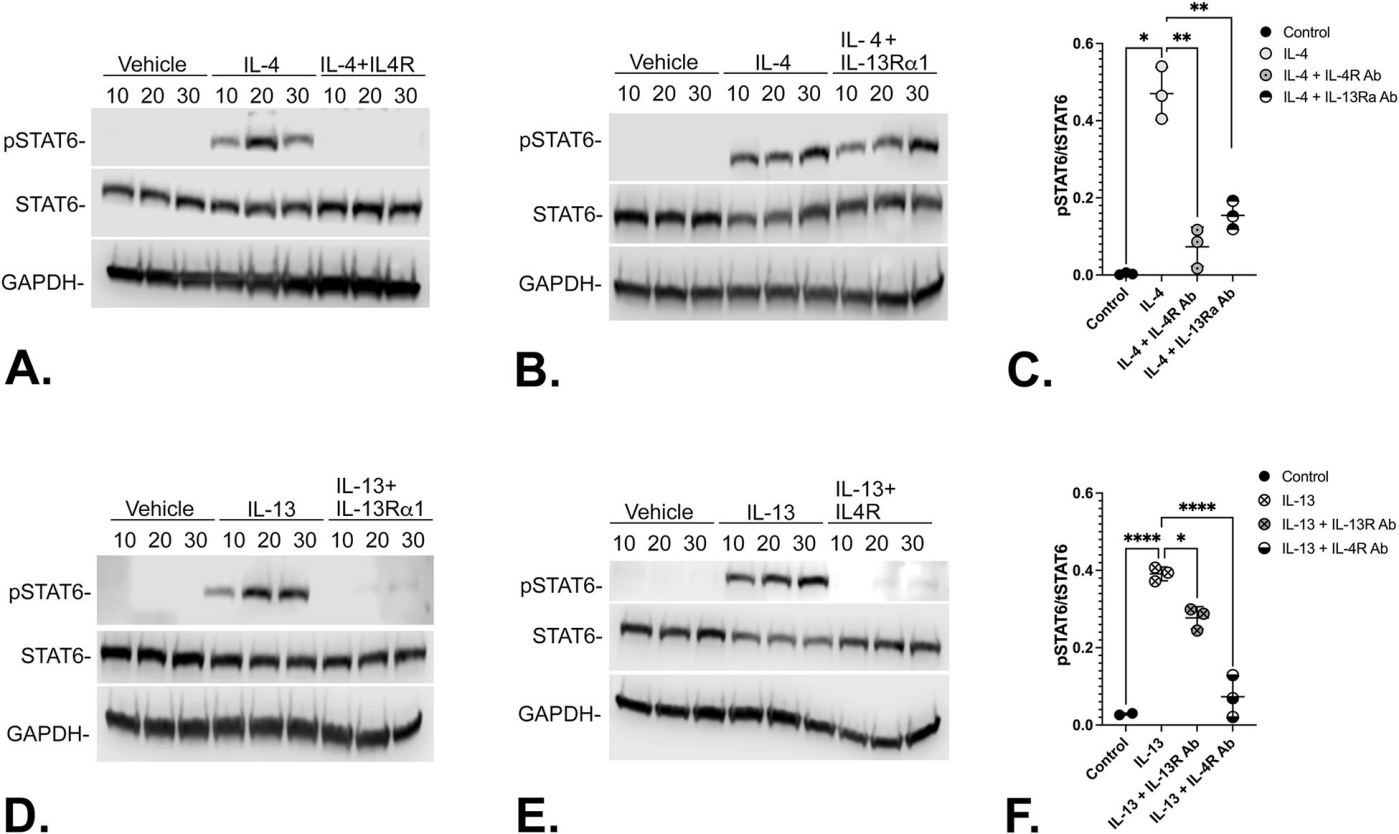
IL-4Rα drives IL-4- and IL-13-mediated signal transduction. N1 immortalized human prostate fibroblasts were treated with vehicle, IL-4 (20ng/ml), or IL-13 (20 ng/m) with or without 2 hr pre-treatment with IL4Rα (IL4R) (400 ng) or IL-13Rα1 (40 ng) antibodies. Both IL-4 (Fig 7A) and IL-13 (Fig 7D) robustly and significantly induced STAT6 phosphoryation compared to vehicle-treated cells. IL-4 stimulation of STAT6 phosphorylation was repressed upon pre-treatment with IL4Rα (**A**) but not IL-13Rα1 (**B**) antibodies, whereas IL-13 stimulation of STAT6 phosphorylation was repressed upon pre-treatment with either IL-13Rα1 (**D**) or IL4Rα (**E**) antibodies. Densitometric data (S7 Fig) from the replicate experiments is graphed in **C** (IL-4) and **F** (IL-13). Significant differences are indicated as * p < .05; ** p < .01; *** p < .001; **** p < .0001.

IL-4 and IL-13 share a common signaling axis that requires the IL-4Rα receptor. IL-4 signals through a IL-4Rα/γc (Type 1) or IL-4Rα/IL-13Rα1 (Type 2) heterodimer, while IL-13 signals solely through the IL-4Rα/IL-13Rα1 Type 2 heterodimer. IL-13Rα2 is thought to act as a decoy receptor to homeostatically sequester IL-13 in normal cells and does not transduce intracellular signaling [26–28]. Signal Transduceer and Activator of Transcription (STAT) proteins comprise a family of six members (STATS 1–6) that are phosphorylated primarily by Janus Kinases (JAKs), in response to IL-4 or IL-13 binding to the heterodimeric receptors described above [29]. In order to determine the relative contribution of IL-4 and IL-13 stimulation to STAT6 phosphorylation in human prostate fibroblasts, N1 immortalized human prostate fibroblasts were treated with vehicle, IL-4, or IL-13 with or without pre-treatment with antibodies against their cognate receptors, IL4Rα or IL-13Rα1, respectively. These studies showed that IL-4 stimulation of STAT6 phosphorylation was repressed upon pre-treatment with antibodies against IL4Rα (Fig 7A and 7C) but not IL-13Rα1 (Fig 7B and 7C), whereas IL-13 stimulation of STAT6 phosphorylation was repressed upon pre-treatment with either IL-13Rα1 (Fig 7D and 7F) or IL4Rα (Fig 7E and 7F) antibodies (S7 Fig). These data suggest downstream signaling medicated by IL-4 may be less suppressible, hence, more robust, than that mediated by IL-13. This is consistent with the ability of IL-4 to signal through both Type 1 and Type 2 heterodimeric receptors.

### IL-4 and IL-13 signal through JAK/STAT to induce collagen 1 and collagen 3 expression

**Fig 8:**
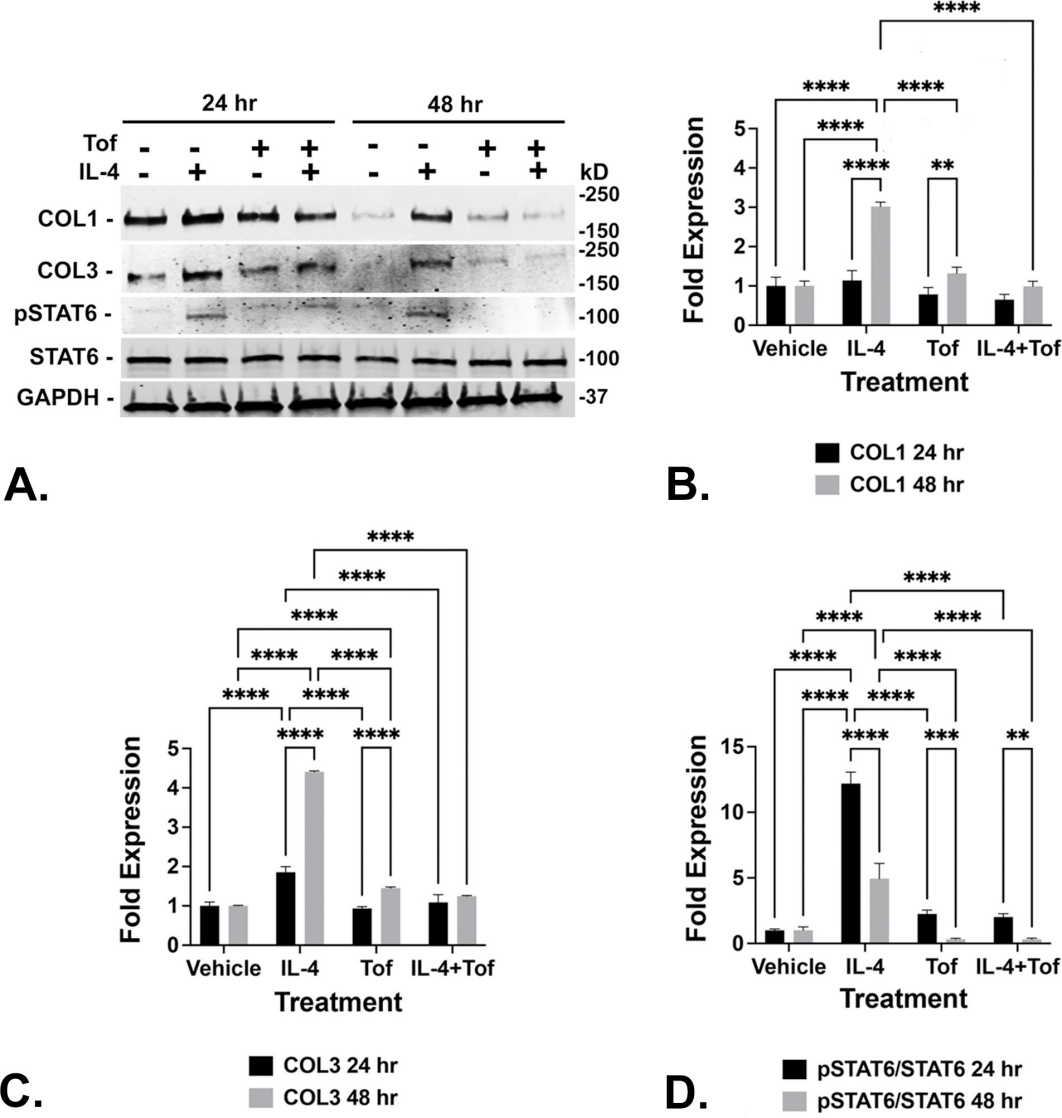
IL-4-mediated induction of collagen 1 and collagen 3 expression is coupled to JAK/STAT signaling. N1 immortalized human prostate fibroblasts were serum-starved for 48 hr then treated with vehicle or IL-4 (20ng/ml) with or without 2 hr pre-treatment with 5um tofacitinib, a JAK2/JAK3 inhibitor, and maintained in serum-free media for an additional 24 or 48 hr. IL-4 significantly induced expression of collagen I (COL1) and collagen 3 (COL3) concurrent with induction of STAT6 phosphorylation, all of which was repressed upon pre-treatment with tofactinib (**A**). Western blots shown in **A** are representative of 3 replicate experiments. Densitometric data from the replicate experiments is graphed in **B** (COL1), **C** (COL3**),** and **D** (pSTAT6/STAT6) (S8 Fig). Significant differences are indicated as * p < .05; ** p < .01; *** p < .001; **** p < .0001.

Upon activation by ligand binding, the IL-4Rα/IL13Rα1 signaling axis activates the transcription of genes encoding pro-fibrotic collagens, which are key components of the ECM and are heavily upregulated in fibrosis (reviewed in [24]). To directly investigate the potential role(s) of JAK/STAT signaling in IL-4 induction of collagen expression, N1 cells were treated with IL-4 with or without pretreatment with 5uM tofacitinib (CP-690550; Xeljanz), a small molecule JAK2/JAK3 inhibitor with low cytoxicity shown to repress IL-4-mediated downstream signaling [30–32]. N1 fibroblasts grown in complete media will express high basal collagen levels likely due to the presence of pro-fibrotic proteins in the serum component of the media. If the complete media is removed, the cells washed, and media replaced with serum-free media, the cells will gradually cease producing collagen (Fig 8A). However, cells grown for 24–48 hr in serum-free media, then treated with IL-4 or IL-13, significantly up-regulate collagen 1 and 3 production over basal levels (Fig 8A–8C). Therefore, collagen 1 and 3 accumulation was concordant with IL-4 treatment, and was reduced or ablated upon pre-treatment with tofactinib (Fig 8). Similarly, phosphorylation of STAT6 is concordant with IL-4-mediated collagen 1 or 3 induction and is robustly maintained by IL-4-treated cells for at least 48 hr in the absence of tofacitinib (Fig 8A–8D) (S8 Fig). These studies show that IL-4 induced expression of collagen 1 and 3 is coupled to JAK/STAT signaling in human prostate fibroblasts.

## Discussion

Aging- and inflammation-associated fibrotic changes in tissue architecture contribute to dysfunction and disease in multiple organ systems [33, 34]. Our studies recently showed that peri-urethral prostatic fibrosis is associated with lower urinary symptoms and urinary voiding dysfunction in men [10, 12]. Fibrosis in the lower urinary tract is associated with aging and likely results from complex interactions between prostate epithelial cells, resident fibroblasts, circulating fibrocytes, and infiltrating inflammation.

Few studies have sought to determine which pro-fibrotics may play predominant roles in lower urinary tract fibrosis. The most studied to-date is TGFß, which has been shown to induce prostatic fibrosis and alter extracellular matrix deposition in mouse models [35]. Some of this activity may be stimulated by an imbalance between estrogen and androgen levels in aging men [36, 37]. A recent study utilized medical record data, patient samples, and in vivo models to evaluate the impact of inflammation, and TNFα in particular, within the context of autoimmune disease, on lower urinary tract disorders. Previous studies from our group showed that pro-inflammatory proteins, including chemokines and interleukins, are secreted by aging prostate fibroblasts, and that one of these, CXCL12, acts as a pro-fibrotic in vitro and in mouse models in vivo [38–40]. The Campisi group showed that many inflammatory mediators, including those mentioned above, are secreted as part of the senescence associate secretory phenotype (SASP) induced by the aging process or, experimentally, by exposure to H_2_0_2_ or irradiation [41]. Several studies have shown that IL-4 and IL-13, which share a common JAK/STAT6 signaling axis, promote fibrillar collagen secretion in multiple organ systems (reviewed in [16], most notably in the lung [17, 18], the kidney [19, 20], and the liver [17, 21].

Immunohistochemical data reported here show that the IL4Rα and IL13Rα1 receptors are up-regulated in peri-urethral prostate tissues in conjunction with high tissue collagen expression and lower urinary tract symptoms (LUTS). Data from the Human Protein Atlas (proteinatlas.org) shows that both IL4Rα and IL13Rα1 are robustly expressed at the RNA and protein levels by immune cells but also by diverse cell types, including fibroblasts and mesenchymal cells, and by diverse tissues, including the human prostate. Combined with the studies cited above showing that IL-4 and IL-13 promote fibrosis in multiple organ systems, the elevated expression of IL4Rα and IL13Rα1 concurrent with prostate tissue fibrosis and lower urinary tract voiding dysunction led to the hypothesis that the IL-4/IL-13 signaling axis may promote prostatic fibrosis as well.

ELISA and immunofluorescence data shown in the current study provide evidence that IL-4 and IL-13 can up-regulate their own expression levels as well as those of their cognate receptors in prostate fibroblasts. This is consistent with data acquired from the study of Th2 cells which shows that Th2 differentiation occurs through a sequential series of events initiated by IL-4 or IL-13 binding to heterodimeric IL-4 receptors. These ligand/receptor interactions activate receptor bound kinases JAKs or Tyk1, which phosphorylate STAT6. pSTAT6 dimerizes and translocates to the nucleus where it promotes its’ own expression and that of the GATA-3 transcription factor. After phosphorylation by diverse kinases, including p38, Akt, and Erk, pGATA-3 translocate to the nucleus where both pSTAT6 and pGATA-3 transcriptionally [42–45] activate IL-4 and IL-13 gene expression [46]. pSTAT6 may also directly activate transcription of the IL-4Rα, COL1A1, and COL1A2 genes [43]. Our data shows that IL-4 and IL-13 self-upregulate their expression as well as that of their cognate receptors, IL-4Rα and IL-13Rα1, in prostate stromal fibroblasts. Both IL-4 and IL-13 promote STAT6 phosphorylation in prostate stromal fibroblasts, and pSTAT6 persists for at least 48 hr post-induction. These combined data suggest a mechanism whereby signaling through the IL-4/IL-13 axis up-regulates and activates the STAT6 transcription factor, which then promotes the expression of IL-4 and IL-13 and their cognate receptors, IL-4Rα and IL-13Rα1, and promotes the expression of collagens 1 and 3, contributing to activated fibroblast accumulation and persistence in the prostate.

Studies intended to examine the mechanism of IL-4/IL-13 mediated activation of STAT6 showed that IL-4 stimulation of STAT6 phosphorylation was repressed upon pre-treatment with antibodies against IL4Rα but not IL-13Rα1, whereas IL-13 stimulation of STAT6 phosphorylation was repressed upon pre-treatment with either IL-13Rα1 or IL4Rα antibodies. These data suggest that IL-4, rather than IL-13, is the ’driver’ of JAK/STAT signaling promoting STAT6 phosphorylation in human prostate fibroblasts, likely due to its ability to signal through both the Type 1 γc/ IL4Rα and Type 2 IL4Rα/ IL-13Rα1 receptor complexes. Tofacitinib, a robust but non-cytotoxic JAK2/JAK3 inhibitor commercially known as Xeljanz, completely repressed IL-4-mediated collagen 1 and collagen 3 expression by these same cells. A recent study showed that mouse models expressing STAT6 developed dorsolateral prostate lobe fibrosis, whereas those lacking STAT6 did not, upon infection with the uropathogenic E. coli strain CP1 [47]. Together these studies confirm the importance of the JAK/STAT axis, and of STAT6-dependent mechanisms, for IL-4-mediated prostate fibroblast activation and ECM accumulation [47].

Although IL-4 and IL-13 clearly up-regulated collagen expression, neither significantly stimulated αSMA expression or adoption of a myofibroblast morphology by prostate stromal fibroblasts. This suggests that activation of the IL-14/IL-13 axis induces prostate fibroblasts to express ECM but not to differentiate completely to myofibroblasts. Fibroblast cell types that secrete ECM but lack αSMA expression or contractile activity have been described as ‘activated fibroblasts’ or ’proto-myofibroblasts’ [48]. More recently, single cell RNA sequencing studies have identified fibroblast cell populations that lack αSMA expression but highly express collagen-encoding genes in mouse models of pulmonary fibrosis [49], and lung and kidney fibrosis [50], and in human skin [51] and human lung fibrosis [52]. Taken together, this data suggests that ECM secreting cells associated with fibrosis may comprise a continuum of fibroblastic cellular phenotypes and multiple sub-populations.

In conclusion, these studies show that IL-4 and IL-13 signal through the IL-4Rα receptor to activate JAK/STAT signaling, thereby promoting their own expression, that of their cognate receptors, and collagens. The concurrent high expression levels of the IL-4Rα and IL-13Rα1 receptors and collagen in peri-urethral tissues from men suffering from LUTS is certainly intriguing. This should be further investigated, along with elucidation of the relative contributions of immune cells and prostate fibroblasts in promoting JAK/STAT signaling and fibrosis, perhaps through using organoid or in vivo mouse models. Taken together, the studies presented here suggest that the IL-4/IL-13 signaling axis is a powerful, but therapeutically targetable, pro-fibrotic mechanism in the lower urinary tract.

## Material and methods

### Institutional review board approval

In all cases, written informed consent was obtained from patients all subjects under protocols approved by the University Institutional Review Board.

### Immunohistochemistry

FFPE tissues were sectioned at five-micron thickness and labeled with IL-4Rα (MAB230, R&D Systems), IL-13Rα1 (AF152, R&D Systems), or IL-13Rα2 (AF146, R&D Systems) antibodies (1:100) using AEC solution and N-Histofine (universal immune-peroxidase polymer, anti-goat/anti-mouse, Nichirei Biosciences INC). IL-4Rα, IL-13Rα1, and IL-13Rα2 expression was detected using the universal immune-peroxidase polymer kit according to manufacturer’s protocol. Appropriate negative (no primary antibody) tissues were stained in parallel. CD8 expression levels were assessed on whole tissue sections using ImmunoMembrane [53], an ImageJ plugin that uses color deconvolution for stain separation and a customized algorithm for cell membrane segmentation. A quantitative score (IM-score, 0–20 points) is generated according to the membrane staining intensity and completeness. Specimens are classified into 0/1+, 2+ or 3+ based on IM-score cut-offs. The classification and membrane segmentation are presented as a pseudo-colored overlay image (S1 Fig). ImmunoMembrane is freely accessible at: http://jvsmicroscope.uta.fi/immunomembrane/.

### Cell culture

N1 cells were derived from prostate transition zone tissue explanted, grown as monolayer cells, and transduced with a recombinant LXSNE6E7 retrovirus [54]. SFT1 cells were derived from prostate transition zone tissue explanted and grown as monolayer cells from a solitary fibrous tumor of the prostate characterized by a NAB2/STAT6 gene fusion [55]. The cells were grown in 5% HIE culture media (Ham’s F-12, 5% FBS, Insulin [5 μg/mL], EGF [10 ng/mL], Hydrocortisone [1 μg/mL], Fungizone [0.5 μg/mL], Gentamicin [0.05 mg/mL]), Plasmocin [0.5 ug/mL]). Prior to treatment, cells were serum starved for 24–48 hr using SF HIE (Ham’s F12, EGF [50 ng/mL], 0.1% BSA, Insulin [5 μg/mL], Transferrin [5 μg/mL], 50 μM sodium selenite, 10 uM 3,3’, 5-triiodo-L-thyronine, HEPES [10mM], Hydrocortisone [1 μg/mL], Fungizone [0.5 μg/mL], Gentamicin [0.05 mg/mL]) Ethanolamine [5mM]. Lung fibroblasts were explanted and grown from lung tissues obtained from patients undergoing thoracic surgery for non-fibrotic lung diseases (normal lung fibroblasts) and were maintained in Dulbecco’s modified Eagle medium (DMEM) with 20% fetal bovine serum (FBS), penicillin, streptomycin and fungizone as described previously [56]. Average cell numbers and standard deviations were calculated.

### Proliferation assays

Proliferation (WST) assays were conducted as previously described [39]. Briefly, cells were seeded at 10000 cells/well in 96 well plates in complete media, washed in 1X PBS and switched to serum-free media (except for a positive control well), treated with increasing doses of IL-4 (0–200 ng/ml) or IL-13 (1–100 ng/ml) for 24 or 48 hours. These studies identified concentrations of IL-4 (20ng/ml) and IL-13 (20 ng/ml) that exhibited robust cellular proliferation. Inhibition studies were conducted by pre-treating cells plated as described above for 2hr with increasing concentrations IL-4Rα (MAB230, R&D Systems) or IL-13Rα1 (AF152, R&D Systems) antibodies followed by stimulation with 20ng/ml IL-4 or IL-13 for 24 hr. Each study was conducted in triplicate and included 9 technical replicates for each condition. Proliferation was assessed by WST assay (Roche, USA, Cat. No.11644807001) (S2 Fig).

### ELISA assays

Cells were cultured as described above and supplemented with vehicle or 20ng/ml IL-4, IL-13, IFN-γ, TNFα, or 4ng/ml TGF-β, for 24 hours with or without 2 hr pre-treatment with 400 ng/ml IL-4Rα (MAB230, R&D Systems) or 40 ng/ml IL-13Rα1 (AF152, R&D Systems) antibodies. The cells were washed with 1XPBS and the cells were cultured for another 24 hrs in serum-free media. 4mls of conditioned media collected from treated cells were spun for 40 minutes at 4,000rpm at 4°C using Amicon Ultracel 3K (Millipore #UFC800324) to concentrate the samples. The samples were then subjected to ELISA human IL-4, IL-13, IFN- γ, TNFα and TGF-β1 immunoassays ELISA Kits (cat. no. D4050, D1300B, DFNAS0, DTA00D, R&D Systems, # MBS761127, Biosource, respectively) (S3 Fig).

### RNA extraction and quantitative real time PCR (qRT-PCR)

Cells were cultured as described above and supplemented with vehicle or 20ng/ml IL-4, IL-13, with or without 2 hr pre-treatment with 400 ng/ml anti-IL-4Rα (MAB230, R&D Systems) or 40 ng/ml anti-IL-13Rα1 (AF152, R&D Systems) antibodies, for 24 hrs. RNA was extracted using Trizol reagent (Invitrogen, Carlsbad, CA), assessed for purity by A260/A280 ratio and quantified using a Nanodrop spectrophotometer. 1 ug of RNA was reverse transcribed using a High Capacity cDNA Reverse Transcription Kit (Applied Biosystems, Carlsbad, CA). qRT-PCR was performed using a QuantStudio 12K Flex Real-Time PCR System, reagents and software (Applied Biosystems, Carlsbad, CA). Reactions were performed in triplicate, including no template controls and amplification of an endogenous control transcript, Larger Ribosomal Protein (RPLPO), to assess template concentration and loading precision. Cycle number to threshold was calculated for each gene by subtracting the average control value from each average experimental value and normalized to RPLPO (loading control) using the Pfaffl method [57]. Molecular probes used were Hs0016400_m1 for COL1α1 and Hs99999902_m1 for RPLPO (Applied Biosystems, Carlsbad, CA) (S4 Fig).

### Sircol assays

Cells were cultured as described above and supplemented with vehicle or 20ng/ml IL-4, IL-13, TNFα, or 4ng/ml TGF-β, for 48 hours with or without 2 hr pre-treatment with 400 ng/ml IL-4Rα (MAB230, R&D Systems) or 40 ng/ml IL-13Rα1 (AF152, R&D Systems) antibodies, then assessed for secreted soluble collagen types I-V using Sircol assay reagents, collagen standards, and procedures as described by the manufacturer (S1000, Life Science) (S4 Fig).

### Immunofluorescence assays

For the detection of COLA1A and aSMA proteins, cells were plated on chamber slides coated with 10 μg/ml fibronectin (Sigma-Aldrich, St. Louis, MO) in complete media washed, switched to serum-free media, and supplemented with vehicle or 20ng/ml IL-4 or IL-13, or 4ng/ml TGF-β, for 48 hours with or without 2 hr pre-treatment with 400 ng/ml IL-4Rα (MAB230, R&D Systems) or 40 ng/ml IL-13Rα1 (AF152, R&D Systems) antibodies. After 48 hr the cells were fixed, permeabilized, and subjected to immunofluorescence as previously described [14], using FITC-conjugated mouse monoclonal anti-αSMA (F3777, Sigma-Aldrich, St. Louis, MO), and biotin conjugated rabbit polyclonal anti-COL1A1 (600-406-103, Rockland Immunochemicals, Gilbertsville, PA), PE-Cy 5 streptavidin (BD Pharmingen San Diego, CA) conjugated secondary antibodies, and control mouse IgG2a (Sigma-Aldrich, St. Louis, MO). Cells were counterstained for 5 min with 1 mg/ml DAPI (Molecular Probes, Eugene, OR) in Tris-Buffered Saline/Tween 20, washed three times for 5 min each with TBST, and mounted in an Aqua-mount (Lerner Laboratories, PA) (S5 Fig)

For the detection of IL-4Rα or IL-13Rα1, cells were grown in 24-well plates in complete media washed, switched to serum-free media, and supplemented with vehicle or 20ng/ml IL-4 or IL-13 for 48 hours. After 48 hr the cells were fixed, permeabilized, blocked using 20% and subjected to immunofluorescence as previously described [14] but using mouse monoclonal IL-4Rα primary antibody (MAB230, R&D Systems) detected with goat anti-mouse IgG H&L (Alexa Fluor 594 (Abacam, ab150116) or goat polyclonal IL-13Rα1 primary antibody (AF152, R&D Systems) detected with donkey anti-goat IgG (H+L) Cross-Adsorbed Secondary Antibody, Alexa Fluor 488 (ThermoFisher A-11055) (S6 Fig).

Photomicrographs of were taken on an Olympus BX53 fluorescence microscope or EVOS Cell Imaging System. Signal density was quantified using Image Pro Plus software.

### Western blot

Cells were treated as described above and lysed in radioimmunoprecipitation assay (RIPA) Buffer. Protein quantification was carried out using Bio-Rad OneStep Bradford reagent and an Elx800 Microplate Reader (Bio-Tek) with Gen5 software. Protein lysates were prepared for electrophoresis as previously described [39]. Membranes were blocked using a 5% BSA in TBS-T solution for detection of phospho antibodies or 5% milk for detection of total antibodies in TBS-T solution for one hour. Primary antibodies incubation was performed using a 5% BSA TBS-T solution with IL-4Rα mouse monoclonal antibody (#25463), IL-13Rα1 goat IgG (#AF146), and anti-IL-13Rα2 goat IgG (#AF152) from R&D Systems, anti-Stat6 (#9362S), Y641 anti-pStat6 (#9361S), Smad3 (#9523), S423/425 pSmad3 (#9520S), anti-Collagen 3 (#30565) and GAPDH (#2118) from Cell Signaling Technologies, and anti-Collagen 1 (#EPR7785) from Abcam. All primary antibodies were incubated overnight at a 1:1000 concentration, except for GAPDH which was used at 1:5000, followed by 3 times washing with TBS-T. Secondary antibody incubations using Horse Radish Peroxidase, HRP, conjugated goat anti-rabbit (Cell Signaling, #7074) or chicken anti-goat IgG HRP conjugated (#SC2953), goat anti-mouse IgG HRP conjugated (#SC2005) from Santa Cruz Biotechnology. Secondary antibodies were used at a 1:5000 concentration for 1 hour at room temperature. Membranes were washed twice with TBS-T and for the detection of HRP-conjugated antibody, immunoblots were quantified and analyzed using the ImageStudio software suite. Immunoblots shown are representative of triplicate experiments (S7 and S8 Figs).

### Statistical analysis

Statistical analysis was accomplished with GraphPad Prism v.9 using 1- or 2-way ANOVA using replicate study data as detailed in the supplemental information). In all tests, p≤.05 was considered statistically significant.

## Supporting information

S1 Fig Fig 1 supporting information. (XLSX)

S2 Fig Fig 2 supporting information. (XLSX)

S3 Fig Fig 3 supporting information. (XLSX)

S4 Fig Fig 4 supporting information. (XLSX)

S5 Fig Fig 5 supporting information. (XLSX)

S6 Fig Fig 6 supporting information. (XLSX)

S7 Fig Fig 7 supporting information. (XLSX)

S8 Fig Fig 8 supporting information. (XLSX)

## References

[1] Laborde EE, McVary KT. Medical management of lower urinary tract symptoms. Rev Urol. 2009;11(Suppl 1):S19–25. Epub 2010/02/04. ; PubMed Central PMCID: PMC2812890. 20126608 PMC2812890

[2] Wei JT, Calhoun E, Jacobsen SJ. Urologic diseases in America project: benign prostatic hyperplasia. J Urol. 2005;173(4):1256–61. Epub 2005/03/11. S0022-5347(05)61064-6 [pii] doi: 10.1097/01.ju.0000155709.37840.fe . 15758764

[3] Kupelian V, Wei JT, O’Leary MP, Kusek JW, Litman HJ, Link CL, et al. Prevalence of lower urinary tract symptoms and effect on quality of life in a racially and ethnically diverse random sample: the Boston Area Community Health (BACH) Survey. Arch Intern Med. 2006;166(21):2381–7. Epub 2006/11/30. 166/21/2381 [pii] doi: 10.1001/archinte.166.21.2381 . 17130393

[4] Irwin DE, Kopp ZS, Agatep B, Milsom I, Abrams P. Worldwide prevalence estimates of lower urinary tract symptoms, overactive bladder, urinary incontinence and bladder outlet obstruction. BJU International. 2011;108(7):1132–8. doi: 10.1111/j.1464-410X.2010.09993.x 21231991

[5] Strope SA, Yang L, Nepple KG, Andriole GL, Owens PL. Population based comparative effectiveness of transurethral resection of the prostate and laser therapy for benign prostatic hyperplasia. J Urol. 2012;187(4):1341–5. doi: 10.1016/j.juro.2011.11.102 ; PubMed Central PMCID: PMC3461307. 22341267 PMC3461307

[6] Theyer G, Kramer G, Assmann I, Sherwood E, Preinfalk W, Marberger M, et al. Phenotypic characterization of infiltrating leukocytes in benign prostatic hyperplasia. Lab Invest. 1992;66(1):96–107. Epub 1992/01/01. . 1370561

[7] Steiner GE, Stix U, Handisurya A, Willheim M, Haitel A, Reithmayr F, et al. Cytokine expression pattern in benign prostatic hyperplasia infiltrating T cells and impact of lymphocytic infiltration on cytokine mRNA profile in prostatic tissue. Lab Invest. 2003;83(8):1131–46. Epub 2003/08/16. doi: 10.1097/01.lab.0000081388.40145.65 . 12920242

[8] Wynn TA. Cellular and molecular mechanisms of fibrosis. The Journal of Pathology. 2008;214(2):199–210. doi: 10.1002/path.2277 18161745 PMC2693329

[9] Torkko KC, Wilson RS, Smith EE, Kusek JW, van Bokhoven A, Lucia MS. Prostate Biopsy Markers of Inflammation are Associated with Risk of Clinical Progression of Benign Prostatic Hyperplasia: Findings from the MTOPS Study. J Urol. 2015;194(2):454–61. Epub 2015/04/02. doi: 10.1016/j.juro.2015.03.103 . 25828974

[10] Ma J, Gharaee-Kermani M, Kunju L, Hollingsworth JM, Adler J, Arruda EM, et al. Prostatic fibrosis is associated with lower urinary tract symptoms. J Urol. 2012;188(4):1375–81. Epub 2012/08/22. doi: 10.1016/j.juro.2012.06.007007S0022-5347(12)03909-2 [pii]. ; PubMed Central PMCID: PMC3485634. 22906651 PMC3485634

[11] Gharaee-Kermani M, Kasina S, Moore BB, Thomas D, Mehra R, Macoska JA. CXC-Type Chemokines Promote Myofibroblast Phenoconversion and Prostatic Fibrosis. PLoS ONE. 2012;7(11):e49278. Epub 2012/11/23. doi: 10.1371/journal.pone.0049278 PONE-D-12-22021 [pii]. . 23173053 PMC3500280

[12] Gharaee-Kermani M, Rodriguez-Nieves JA, Mehra R, Vezina CA, Sarma AV, Macoska JA. Obesity-induced diabetes and lower urinary tract fibrosis promote urinary voiding dysfunction in a mouse model. Prostate. 2013;73(10):1123–33. Epub 2013/03/28. doi: 10.1002/pros.22662 ; PubMed Central PMCID: PMC5512573. 23532836 PMC5512573

[13] Rodriguez-Nieves JA, Macoska JA. Prostatic fibrosis, lower urinary tract symptoms, and BPH. Nature reviews Urology. 2013;10(9):546–50. doi: 10.1038/nrurol.2013.149 . 23857178 PMC5625295

[14] Rodriguez-Nieves JA, Patalano SC, Almanza D, Gharaee-Kermani M, Macoska JA. CXCL12/CXCR4 Axis Activation Mediates Prostate Myofibroblast Phenoconversion through Non-Canonical EGFR/MEK/ERK Signaling. PLoS One. 2016;11(7):e0159490. doi: 10.1371/journal.pone.0159490 ; PubMed Central PMCID: PMC4951124. 27434301 PMC4951124

[15] Patalano S, Rodriguez-Nieves J, Colaneri C, Cotellessa J, Almanza D, Zhilin-Roth A, et al. CXCL12/CXCR4-Mediated Procollagen Secretion Is Coupled To Cullin-RING Ubiquitin Ligase Activation. Sci Rep. 2018;8(1):3499. Epub 2018/02/24. doi: 10.1038/s41598-018-21506-7 ; PubMed Central PMCID: PMC5823879. 29472636 PMC5823879

[16] Gieseck RL 3rd, Wilson MS, Wynn TA. Type 2 immunity in tissue repair and fibrosis. Nat Rev Immunol. 2018;18(1):62–76. Epub 2017/08/31. doi: 10.1038/nri.2017.90 . 28853443

[17] Weng SY, Wang X, Vijayan S, Tang Y, Kim YO, Padberg K, et al. IL-4 Receptor Alpha Signaling through Macrophages Differentially Regulates Liver Fibrosis Progression and Reversal. EBioMedicine. 2018;29:92–103. Epub 2018/02/22. doi: 10.1016/j.ebiom.2018.01.028 ; PubMed Central PMCID: PMC5925448. 29463471 PMC5925448

[18] Montero P, Milara J, Roger I, Cortijo J. Role of JAK/STAT in Interstitial Lung Diseases; Molecular and Cellular Mechanisms. Int J Mol Sci. 2021;22(12). Epub 2021/07/03. doi: 10.3390/ijms22126211 ; PubMed Central PMCID: PMC8226626. 34207510 PMC8226626

[19] Liang H, Zhang Z, Yan J, Wang Y, Hu Z, Mitch WE, et al. The IL-4 receptor alpha has a critical role in bone marrow-derived fibroblast activation and renal fibrosis. Kidney Int. 2017;92(6):1433–43. Epub 2017/07/26. doi: 10.1016/j.kint.2017.04.021 ; PubMed Central PMCID: PMC5696054. 28739140 PMC5696054

[20] Jiao B, An C, Tran M, Du H, Wang P, Zhou D, et al. Pharmacological Inhibition of STAT6 Ameliorates Myeloid Fibroblast Activation and Alternative Macrophage Polarization in Renal Fibrosis. Front Immunol. 2021;12:735014. Epub 2021/09/14. doi: 10.3389/fimmu.2021.735014 ; PubMed Central PMCID: PMC8426438. 34512669 PMC8426438

[21] Chiaramonte MG, Donaldson DD, Cheever AW, Wynn TA. An IL-13 inhibitor blocks the development of hepatic fibrosis during a T-helper type 2-dominated inflammatory response. J Clin Invest. 1999;104(6):777–85. Epub 1999/09/24. doi: 10.1172/JCI7325 ; PubMed Central PMCID: PMC408441. 10491413 PMC408441

[22] Macoska JA UK, Leverson G, McVary KT, Ricke WA. Prostate Transition Zone Fibrosis in Men who Who Failed Doxazosin, Finasteride, or Combination Therapy in the Medical Therapy of Prostatic Symptoms (MTOPS) Submitted. 2019.

[23] Leonard WJ, Lin JX, O’Shea JJ. The gammac Family of Cytokines: Basic Biology to Therapeutic Ramifications. Immunity. 2019;50(4):832–50. Epub 2019/04/18. doi: 10.1016/j.immuni.2019.03.028 . 30995502

[24] Mueller TD, Zhang JL, Sebald W, Duschl A. Structure, binding, and antagonists in the IL-4/IL-13 receptor system. Biochim Biophys Acta. 2002;1592(3):237–50. Epub 2002/11/08. doi: 10.1016/s0167-4889(02)00318-x . 12421669

[25] Borthwick LA, Wynn TA, Fisher AJ. Cytokine mediated tissue fibrosis. Biochim Biophys Acta. 2013;1832(7):1049–60. Epub 2012/10/11. doi: 10.1016/j.bbadis.2012.09.014 ; PubMed Central PMCID: PMC3787896. 23046809 PMC3787896

[26] LaPorte SL, Juo ZS, Vaclavikova J, Colf LA, Qi X, Heller NM, et al. Molecular and structural basis of cytokine receptor pleiotropy in the interleukin-4/13 system. Cell. 2008;132(2):259–72. Epub 2008/02/05. doi: 10.1016/j.cell.2007.12.030 ; PubMed Central PMCID: PMC2265076. 18243101 PMC2265076

[27] Ul-Haq Z, Naz S, Mesaik MA. Interleukin-4 receptor signaling and its binding mechanism: A therapeutic insight from inhibitors tool box. Cytokine Growth Factor Rev. 2016;32:3–15. Epub 2016/05/12. doi: 10.1016/j.cytogfr.2016.04.002 . 27165851

[28] Wills-Karp M, Finkelman FD. Untangling the complex web of IL-4- and IL-13-mediated signaling pathways. Sci Signal. 2008;1(51):pe55. Epub 2008/12/26. doi: 10.1126/scisignal.1.51.pe55 ; PubMed Central PMCID: PMC4446705. 19109238 PMC4446705

[29] Bharadwaj U, Kasembeli MM, Robinson P, Tweardy DJ. Targeting Janus Kinases and Signal Transducer and Activator of Transcription 3 to Treat Inflammation, Fibrosis, and Cancer: Rationale, Progress, and Caution. Pharmacol Rev. 2020;72(2):486–526. Epub 2020/03/22. doi: 10.1124/pr.119.018440 ; PubMed Central PMCID: PMC7300325 covering the use of TTI-101, a small-molecule inhibitor of STAT3 cited in this review. These patents are exclusively licensed to Tvardi Therapeutics, which was founded and is co-owned by D.J.T. 32198236 PMC7300325

[30] Wu J, Patmore DM, Jousma E, Eaves DW, Breving K, Patel AV, et al. EGFR-STAT3 signaling promotes formation of malignant peripheral nerve sheath tumors. Oncogene. 2014;33(2):173–80. Epub 2013/01/16. doi: 10.1038/onc.2012.579 ; PubMed Central PMCID: PMC3923530. 23318430 PMC3923530

[31] Angelini J, Talotta R, Roncato R, Fornasier G, Barbiero G, Dal Cin L, et al. JAK-Inhibitors for the Treatment of Rheumatoid Arthritis: A Focus on the Present and an Outlook on the Future. Biomolecules. 2020;10(7). Epub 2020/07/09. doi: 10.3390/biom10071002 ; PubMed Central PMCID: PMC7408575. 32635659 PMC7408575

[32] Nappo G, Handle F, Santer FR, McNeill RV, Seed RI, Collins AT, et al. The immunosuppressive cytokine interleukin-4 increases the clonogenic potential of prostate stem-like cells by activation of STAT6 signalling. Oncogenesis. 2017;6(5):e342. Epub 2017/05/30. doi: 10.1038/oncsis.2017.23 ; PubMed Central PMCID: PMC5523058. 28553931 PMC5523058

[33] Rieder F, Fiocchi C. Intestinal fibrosis in IBD—a dynamic, multifactorial process. Nature Reviews Gastroenterology & Hepatology. 2009;6(4):228–35. doi: 10.1038/nrgastro.2009.31 19347014

[34] Goldacre MJ. Demography of aging and the epidemiology of gastrointestinal disorders in the elderly. Best Practice & Research Clinical Gastroenterology. 2009;23(6):793–804. doi: 10.1016/j.bpg.2009.10.008 19942158

[35] Barron DA, Strand DW, Ressler SJ, Dang TD, Hayward SW, Yang F, et al. TGF-beta1 induces an age-dependent inflammation of nerve ganglia and fibroplasia in the prostate gland stroma of a novel transgenic mouse. PLoS ONE. 2010;5(10):e13751. Epub 2010/11/10. doi: 10.1371/journal.pone.0013751 ; PubMed Central PMCID: PMC2966419. 21060787 PMC2966419

[36] Yang Y, Sheng J, Hu S, Cui Y, Xiao J, Yu W, et al. Estrogen and G protein-coupled estrogen receptor accelerate the progression of benign prostatic hyperplasia by inducing prostatic fibrosis. Cell Death Dis. 2022;13(6):533. Epub 2022/06/08. doi: 10.1038/s41419-022-04979-3 ; PubMed Central PMCID: PMC9174491. 35672281 PMC9174491

[37] Kajiwara S, Ishii K, Sasaki T, Kato M, Nishikawa K, Kanda H, et al. Castration-induced stromal remodeling disrupts the reconstituted prostate epithelial structure. Lab Invest. 2020;100(5):670–81. Epub 2019/12/21. doi: 10.1038/s41374-019-0352-4 . 31857695

[38] Begley LA, Kasina S, MacDonald J, Macoska JA. The inflammatory microenvironment of the aging prostate facilitates cellular proliferation and hypertrophy. Cytokine. 2008;43(2):194–9. Epub 2008/06/24. S1043-4666(08)00143-9 [pii] doi: 10.1016/j.cyto.2008.05.012 ; PubMed Central PMCID: PMC2538565. 18572414 PMC2538565

[39] Begley L, Monteleon C, Shah RB, Macdonald JW, Macoska JA. CXCL12 overexpression and secretion by aging fibroblasts enhance human prostate epithelial proliferation in vitro. Aging Cell. 2005;4(6):291–8. Epub 2005/11/23. ACE173 [pii] doi: 10.1111/j.1474-9726.2005.00173.x . 16300481

[40] Macoska JA WZ-Y, Virta J, Zacharias N, Bjorling DE. Inhibition of the CXCL12/CXCR4 Axis Prevents Urethral Collagen Accumulation and Lower Urinary Tract Dysfunction In Vivo. The Prostate. 2019;In Press. 10.1002/pros.23781 PMC7269149 30811623

[41] Coppe JP, Patil CK, Rodier F, Sun Y, Munoz DP, Goldstein J, et al. Senescence-associated secretory phenotypes reveal cell-nonautonomous functions of oncogenic RAS and the p53 tumor suppressor. PLoS Biol. 2008;6(12):2853–68. Epub 2008/12/05. 08-PLBI-RA-2566 [pii] doi: 10.1371/journal.pbio.0060301 ; PubMed Central PMCID: PMC2592359. 19053174 PMC2592359

[42] Hebenstreit D, Wirnsberger G, Horejs-Hoeck J, Duschl A. Signaling mechanisms, interaction partners, and target genes of STAT6. Cytokine Growth Factor Rev. 2006;17(3):173–88. Epub 2006/03/17. doi: 10.1016/j.cytogfr.2006.01.004 . 16540365

[43] Maneechotesuwan K, Xin Y, Ito K, Jazrawi E, Lee KY, Usmani OS, et al. Regulation of Th2 cytokine genes by p38 MAPK-mediated phosphorylation of GATA-3. J Immunol. 2007;178(4):2491–8. Epub 2007/02/06. doi: 10.4049/jimmunol.178.4.2491 . 17277157

[44] Hosokawa H, Tanaka T, Endo Y, Kato M, Shinoda K, Suzuki A, et al. Akt1-mediated Gata3 phosphorylation controls the repression of IFNgamma in memory-type Th2 cells. Nat Commun. 2016;7:11289. Epub 2016/04/08. doi: 10.1038/ncomms11289 ; PubMed Central PMCID: PMC4829694. 27053161 PMC4829694

[45] Liberman AC, Druker J, Refojo D, Holsboer F, Arzt E. Glucocorticoids inhibit GATA-3 phosphorylation and activity in T cells. FASEB J. 2009;23(5):1558–71. Epub 2009/01/07. doi: 10.1096/fj.08-121236 . 19124555

[46] Bao K, Reinhardt RL. The differential expression of IL-4 and IL-13 and its impact on type-2 immunity. Cytokine. 2015;75(1):25–37. Epub 2015/06/16. doi: 10.1016/j.cyto.2015.05.008 ; PubMed Central PMCID: PMC5118948. 26073683 PMC5118948

[47] Bell-Cohn A, Mazur DJ, Hall CC, Schaeffer AJ, Thumbikat P. Uropathogenic Escherichia coli-Induced Fibrosis, leading to Lower Urinary Tract Symptoms, is associated with Type-2 cytokine signaling. Am J Physiol Renal Physiol. 2019. Epub 2019/01/10. doi: 10.1152/ajprenal.00222.2018 . 30623726 PMC6483034

[48] Hinz B, Phan SH, Thannickal VJ, Galli A, Bochaton-Piallat ML, Gabbiani G. The myofibroblast: one function, multiple origins. Am J Pathol. 2007;170(6):1807–16. doi: 10.2353/ajpath.2007.070112 ; PubMed Central PMCID: PMC1899462. 17525249 PMC1899462

[49] Xie T, Wang Y, Deng N, Huang G, Taghavifar F, Geng Y, et al. Single-Cell Deconvolution of Fibroblast Heterogeneity in Mouse Pulmonary Fibrosis. Cell Rep. 2018;22(13):3625–40. Epub 2018/03/29. doi: 10.1016/j.celrep.2018.03.010 ; PubMed Central PMCID: PMC5908225. 29590628 PMC5908225

[50] Sun KH, Chang Y, Reed NI, Sheppard D. alpha-Smooth muscle actin is an inconsistent marker of fibroblasts responsible for force-dependent TGFbeta activation or collagen production across multiple models of organ fibrosis. Am J Physiol Lung Cell Mol Physiol. 2016;310(9):L824–36. Epub 2016/03/06. doi: 10.1152/ajplung.00350.2015 ; PubMed Central PMCID: PMC4867351. 26944089 PMC4867351

[51] Vorstandlechner V, Laggner M, Kalinina P, Haslik W, Radtke C, Shaw L, et al. Deciphering the functional heterogeneity of skin fibroblasts using single-cell RNA sequencing. FASEB J. 2020;34(3):3677–92. Epub 2020/01/14. doi: 10.1096/fj.201902001RR . 31930613

[52] Reyfman PA, Walter JM, Joshi N, Anekalla KR, McQuattie-Pimentel AC, Chiu S, et al. Single-Cell Transcriptomic Analysis of Human Lung Provides Insights into the Pathobiology of Pulmonary Fibrosis. Am J Respir Crit Care Med. 2019;199(12):1517–36. Epub 2018/12/18. doi: 10.1164/rccm.201712-2410OC ; PubMed Central PMCID: PMC6580683. 30554520 PMC6580683

[53] Tuominen VJ, Ruotoistenmaki S, Viitanen A, Jumppanen M, Isola J. ImmunoRatio: a publicly available web application for quantitative image analysis of estrogen receptor (ER), progesterone receptor (PR), and Ki-67. Breast Cancer Res. 2010;12(4):R56. Epub 2010/07/29. doi: 10.1186/bcr2615 ; PubMed Central PMCID: PMC2949645. 20663194 PMC2949645

[54] Begley L, Keeney D, Beheshti B, Squire JA, Kant R, Chaib H, et al. Concordant copy number and transcriptional activity of genes mapping to derivative chromosomes 8 during cellular immortalization in vitro. Genes Chromosomes Cancer. 2006;45(2):136–46. Epub 2005/10/20. doi: 10.1002/gcc.20274 . 16235240

[55] Gharaee-Kermani M, Mehra R, Robinson DR, Wei JT, Macoska JA. Complex Cellular Composition of Solitary Fibrous Tumor of the Prostate. Am J Pathol. 2014. doi: 10.1016/j.ajpath.2013.11.024 . 24434011 PMC3936322

[56] White ES, Thannickal VJ, Carskadon SL, Dickie EG, Livant DL, Markwart S, et al. Integrin alpha4beta1 regulates migration across basement membranes by lung fibroblasts: a role for phosphatase and tensin homologue deleted on chromosome 10. Am J Respir Crit Care Med. 2003;168(4):436–42. doi: 10.1164/rccm.200301-041OC ; PubMed Central PMCID: PMC1997294. 12791582 PMC1997294

[57] Pfaffl MW. A new mathematical model for relative quantification in real-time RT-PCR. Nucleic Acids Res. 2001;29(9):e45. Epub 2001/05/09. doi: 10.1093/nar/29.9.e45 ; PubMed Central PMCID: PMC55695. 11328886 PMC55695

